# Measurement invariance of the center for epidemiological studies depression scale (CES-D) among chinese and dutch elderly

**DOI:** 10.1186/1471-2288-11-74

**Published:** 2011-05-20

**Authors:** Baoshan Zhang, Marjolein Fokkema, Pim Cuijpers, Juan Li, Niels Smits, Aartjan Beekman

**Affiliations:** 1 Center on Aging Psychology, Key Laboratory of Mental Health, Institute of Psychology, Chinese Academy of Sciences, Beijing, China; 2 Department of Clinical Psychology, Faculty of Psychology and Education, Vrije Universiteit Amsterdam, The Netherlands; 3 Institute for Research in Extramural Medicine and Department of Psychiatry, Vrije Universiteit, Amsterdam, The Netherlands

## Abstract

**Background:**

Although previous studies using non- elderly groups have assessed the factorial invariance of the Center for Epidemiological Studies Depression Scale (CES-D) across different groups with the same social-cultural backgrounds, few studies have tested the factorial invariance of the CES-D across two elderly groups from countries with different social cultures. The purposes of this study were to examine the factorial structure of the CES-D, and test its measurement invariance across two different national elderly populations.

**Methods:**

A total of 6806 elderly adults from China (n = 4903) and the Netherlands (n = 1903) were included in the final sample. The CES-D was assessed in both samples. Three strategies were used in the data analysis procedure. First, a confirmatory factor analysis (CFA) was carried out to determine the factor structures of the CES-D that best fitted the two samples. Second, the best fitting model was incorporated into a multi-group CFA model to test measurement invariance of the CES-D across the two population groups. Third, latent mean differences between the two groups were tested.

**Results:**

The results of confirmatory factor analysis (CFA) showed: 1) in both samples, Radloff's four-factor model resulted in a significantly better fit and the four dimensions (somatic complaints, depressed affect, positive affect, and interpersonal problems) of the CES-D seem to be the most informative in assessing depressive symptoms compared to the single-, three-, and the second-order factor models; and 2) the factorial structure was invariant across the populations under study. However, only partial scalar and uniqueness invariance of the CES-D items was supported. Latent means in the partial invariant model were lower for the Dutch sample, compared to the Chinese sample.

**Conclusions:**

Our findings provide evidence of a valid factorial structure of the CES-D that could be applied to elderly populations from both China and the Netherlands, producing a meaningful comparison of total scores between the two elderly groups. However, for some specific factors and items, caution is required when comparing the depressive symptoms between Chinese and Dutch elderly groups.

## Background

### Center for Epidemiological Studies Depression Scale and Its Factor Structures

Depression is considered an important public health problem because of its relatively high prevalence in the general population [1] and its empirically established association with suicidal attempts, prolonged social isolation, and poor physical health [2-4]. In addition, depression has a profound impact on well-being, daily functioning and (excessive) use of health services [5]. The essential components of depression include depressed mood, feelings of guilt and worthlessness, feelings of hopelessness and helplessness, loss of appetite, psychomotor retardation, as well as sleep disturbance [4]. By selecting items from other instruments that reflected these components, Radloff (1977) designed a 20-item inventory, the 'Center for Epidemiological Studies Depression Scale (CES-D)', to assess depressive symptoms in a community-based population [4]. Since its publication in 1977, the scale has become one of the most frequently used self-report depressive symptom scales and has been shown to have good psychometric properties, including desirable internal consistency, good test-retest reliability, as well as high correlations with significant life events and clinical diagnosis of depression [1,4,6]. In a series of studies using the data from Longitudinal Aging Study Amsterdam (LASA), Beekman et al. tested the measurement properties of the Dutch version of the CES-D and found that the psychometric properties of the scale were satisfactory in these studies [7,8].

In the initial report, Radloff (1977) examined the factor structure of the CES-D using principal components analysis with varimax rotation and identified four factors, including Depressed Affect, Positive Affect, Somatic Symptoms/Retarded Activity and Interpersonal problems [4]. Following Radloff's (1977) factor analytic procedures, this four-factor structure of the CES-D has been extensively replicated and widely accepted in subsequent studies [9,10]. However, Radloff (1977) argues against undue emphasis on separate factors and suggests using a simple total score to measure depressive symptomatology, so multifactorial models could be more justified if they include a higher order construct. Therefore, various authors have proposed an alternative higher order factor structure of the CES-D [1,11], in which the four first-order factors are considered to be dependent on a single second-order factor for depression. The study conducted by Gonçalves and Fagulha (2004) revealed a reasonable fit of this four-factor model with a second-order factor [12].

However, there are some inconsistencies concerning the factor structure of the CES-D in the research literature. A three-factor solution is another widely accepted model. Using the data of Hispanic Health and Nutrition Examination Survey (Hispanic HANES), Guarnaccia et al. (1989) identified a three-factor model (i.e., Affect/Somatic, Interpersonal, and Positive), with somatic symptoms and depressive affect combined as one factor rather than two distinct factors [13]. Other studies also found support for the three-factor structure of the CES-D [14,15].

### CES-D and Elderly Populations

The elderly population represents a fast growing segment in most societies. Although there is no direct causal relationship between age and depression (a higher age may be associated with more illness, and physical illness may be associated with depression [16]), depressive symptoms are often observed in elderly populations [17]. In concordance with this fact, more and more researchers have focused their interests on the area of geriatric depression, and the CES-D has been widely used to measure depression among the elderly population. An extensive body of research has established that scores on the CES-D correlate significantly with other measures of depression (e.g., Geriatric Depression Scale) in the elderly population [18,19]. Although most of the initial work on the CES-D was conducted with the general population, measures of depression are increasingly used in research with elderly adults who are at socioeconomic and other types of risk. A large number of studies using the CES-D have demonstrated significant differences concerning depression between males and females [20,21], poor and wealthy [22], whites and minority groups [23], as well as population groups from Eastern and Western cultures [15]. In most studies, the main interests focused on mean group differences. However, the inter-group validity of the CES-D should be established before we can ascertain whether these mean group differences are meaningful. That is, if a difference of the CES-D scores between two group means is observed, one would want to be sure it is caused by a difference in the latent construct of interest, not by response bias. Therefore, although the CES-D was found to be a reliable and valid instrument for measuring depressive symptoms, it remains an empirical question whether it measures the same construct in different populations. Moreover, the subsequent question of whether this instrument measures the construct in the same way, should also be addressed to enable valid comparisons of observed scores.

### Cultural differences in depression

In addition, it is well known that social and cultural differences between countries may result in disagreement about the definitions of depressive symptoms. For example, in the Eastern culture, especially for the Chinese, strongly positive affects run counter to and emotional controls are highly valued by the social culture. Consistent with this notion, previous studies have demonstrated that the Chinese are more likely to value low arousal positive affect (e.g., calmness) than the Western participants, whereas Western participants value high arousal affect (e.g., excitement) more than the Chinese participants [24]. In addition, compared to Western culture, because of the threat to close relationships and the stigma surrounding mental illness, expression of depressed affect is more likely to be devalued by the Eastern collective cultures [25,26]. As a result of these cultural differences, in non-Western countries (e.g., China), compared to Western countries, somatic symptoms tend to be emphasized [27,28], whereas psychological symptoms such as self deprecation, suicidal ideation, and depressed mood are less common [27,29]. Furthermore, when comparing patient groups, Western patients present with more complaints of depressed mood than Chinese patients [30]. Given this evidence, some depressive symptoms may be under- or over- reported in Eastern countries when applying standard measures that have been primarily validated among Western countries.

### Measurement invariance

Vandenberg & Lance (2000) suggested that when using the same measure in two different (cultural) groups, measurement invariance should be established to ascertain whether a given set of measures taps a particular latent construct (such as depression) similarly across groups, so that meaningful comparisons between groups can be made [31]. Put simply, measurement invariance indicates that the instrument measures the same construct in the same way across populations or groups [32].

Although previous studies using non-elderly groups have assessed the factorial invariance of the CES-D across different immigrant [33,34], ethnic [35] and socioeconomic status groups [36], most groups used in these studies were selected from the same social-cultural backgrounds. Few studies have tested the factorial invariance of the CES-D across two elderly groups from countries with different social cultures (e.g., countries with typical characteristics of Eastern social cultures vs. countries with typical characteristics of Western social cultures).

By using two elderly groups recruited from China and the Netherlands, the current study attempted to test the measurement invariance of the CES-D to ascertain whether these two socially and culturally contrasting groups interpret the constructs underlying the CES-D items in a conceptually similar manner. First, various hypothesized factorial structures of the CES-D are tested (i.e., single factor, three factors, four factors, and second-order factor). Second, the equality across the two samples of the parameters characterizing the relationship between the items of the CES-D and the underlying latent constructs are tested. Third, when the measurement invariance was established, the latent mean differences between the two groups were assessed.

## Method

### Participants

The Chinese sample was from the National Survey of Mental Health among Chinese Elderly Adults, conducted by the Institute of Psychology, Chinese Academy of Science. The target population consisted of elderly adults aged 55 and over, residing in the major districts of the Chinese mainland. Data was collected in 2007-2008 through a multistage area national probability sample. A total of 4,903 elderly Chinese adults were included in our final analysis. Of all the participants in the Chinese sample, 2,415 were male (mean age = 67.35 ± 8.21 years) and 2,464 were female (mean age = 66.36 ± 7.97); 24 did not report their gender.

The Dutch sample was from the NESTOR (The Netherlands Program for Research on Aging) Study on Living Arrangements and Social Networks (LSN) which was continued in the Longitudinal Aging Study Amsterdam (LASA), an ongoing longitudinal study with secondary studies on various topics. The target population consisted of elderly adults aged 55 to 85 years of age, residing in urban and rural areas in the West, North-East and South of the Netherlands. Data was used from the fifth wave of the LASA study, which was collected in 2005-2006. A total of 1,903 elderly adults were included in the Dutch sample. Of all participants in the Dutch sample, 853 were male (mean age = 70.43 ± 8.76 years) and 1,050 were female (mean age = 71.79 ± 9.41). A detailed discussion of the LASA sample was provided in the paper of Deeg et al. published in 2002 [37].

In both samples, less than 1% of itemscores were missing. This amount of missing data can be deemed inconsequential [38]. As a result, all available data was used for calculation of covariances and means.

Both surveys were performed with the approval of two appropriate ethics committees. For the Chinese sample, the survey was approved by the ethics committee of the Institute of Psychology. Written informed consent was obtained from each participant. For the Dutch sample, informed consent was obtained at the beginning of the study, in accordance with legal requirements in the Netherlands. Ethical aspects of the research procedures were approved in 1992 by the committee on ethics of research in humans of the Faculty of Medicine of the Vrije Universiteit.

### Measurement

The Center for Epidemiological Studies Depression Scale (CES-D) was used to measure levels of depressive symptoms among elderly participants. The CES-D consists of 16 negative affect and 4 positive affect items, such as "I felt depressed", "I felt lonely", and "I was happy". Participants were asked about the number of days on which they experienced depressive symptoms during the previous week. Each item was accompanied by a standard four-point Likert scale of potential responses: 1 = none, 2 = one or two days a week, 3 = three or four days per week, and 4 = five days or more per week. Higher scores on the CES-D indicate more depressive symptoms [4]. In the scale, four items that describe positive affects were reversed before conducting our analysis. The Chinese version of this scale has been validated [39] and extensively used in studies of Chinese adults. The measurement properties of the Dutch version of the CES-D were tested by Beekman et al using the LASA data [7,8]. The Chinese and Dutch versions of the CES-D, which were used for the current study, are presented in the Appendix.

Radloff (1977) identified four factors in the CES-D in the general population, including somatic complaints, depressed affect, positive affect and interpersonal problems. Items associated with the four factors are listed in table 1. This four-factor model was extensively replicated and widely accepted in previous studies.

**Table 1:** Factors of the CES-D and related items

Somatic complaints	Depressive affect	Positive affect	Interpersonal problems
Bothered	Blues	Good	Unfriendly
Appetite	Depressed	Hopeful	Dislike
Mind	Lonely	Happy	
Effort	Crying	Enjoyed	
Sleep	Sad		
Talk	Failure		
Get Going	Fearful		

Four competing models were tested in the present study: a one, three and four-factor model, and an additional second-order factor model. In the second-order model, the four factors suggested by Radloff (1977) were considered to be dependent on a single second-order factor. The three-factor (i.e., Affect/Somatic, Interpersonal, and Positive) model combines somatic complaints and depressive affect factors and was examined in a number of earlier studies [13]. A one-factor model was frequently tested in previous studies [11,40]. The total score of the CES-D items is generally used as an indicator of depression, which suggests a unidimensional structure. Although this model is not supported by most factor analytic studies, the current study also took the single factor structure as a competing model.

### Analysis

Confirmatory factor analysis (CFA) with maximum likelihood estimation, using LISREL 8.70 [41], was employed to assess how well the data fit the competing (or the nested) models. There were three main aims of this study. First, a CFA was carried out to determine the factor structures of the CES-D that best fitted the Chinese and Dutch datasets, respectively.

Second, after the best fitting model was determined for each sample, it was incorporated into a multi-group CFA model to test measurement invariance of the CES-D across the two population groups. Measurement invariance can be established by running a multi-group analysis of the factor structure underlying the data of these two groups [42]. Traditionally, four nested models are tested in the following order: configural invariance, metric invariance, scalar invariance, and uniqueness invariance [31,43]. In the configural invariance model, the same factorial structures (i.e., the same pattern of free and fixed factor loadings) are specified for each sample, and no equality constraints are imposed on the intercepts, factor loadings, and residual variances across samples; factor means are fixed to zero in both samples. In the metric invariance model, factor loadings are constrained to be equal across samples. In the scalar invariance model, both intercepts and loadings are constrained to be equal across groups. Scalar invariance should be obtained to ascertain that observed scores are the same across groups for identical factor scores [44,45]. Finally, in the uniqueness invariance model, the uniquenesses associated with each item are constrained to be equal across the two groups when factor loadings and intercepts are constrained to invariance.

Third, partial invariance of each model was allowed to refine the structural models [43], as invariance restrictions may hold for some but not all items across samples. Relaxing invariance constraints from the non-invariant items could control for partial measurement inequivalence [43,46]. Values of χ2, RMSEA, and CFI in the LISREL output were studied to determine which item parameters showed a lack of invariance. Equality restrictions of item parameters showing the highest changes in the above indices were lifted until model fit was adequate.

Fourth, following the assessment of measurement invariance, latent mean differences for each latent construct were tested,. In the analysis, latent mean values were fixed to zero in the Chinese group, and freely estimated for the Dutch group. Based on the difference from zero of the latent mean in the Dutch group, latent means can be compared. Statistical significance of the difference can be based on the *t*-statistic of the estimated latent mean in the Dutch group [46]. However, test statistics are expected to be large and significant with the sample sizes in the current study. Consequently, effect sizes for the differences between latent means, *d *values, were calculated according to the guidelines of Hancock (2001) [47].

To evaluate model fit in the current study, Minimum Fit Function Chi-Square χ2, df, RMSEA (root mean square error of approximation, values lower than .08 are accepted), NNFI (Non-Normed Fit Index, values greater than .90 are accepted), CFI (comparative fit index, values greater than .90 are accepted), and AIC (Akaike information criterion, a helpful index for comparing models that are not nested; lower values indicate a better model fit) values are reported. Among these indices, differences of χ2 and df statistics between two invariant models are frequently used to determine whether models' invariance constraints are likely to hold or not. However, a number of problems result from using the χ2 value to evaluate model fit: the χ2 (or Δχ2) is sensitive to minor departures from multivariate normality and is nearly always large and statistically significant with complex models and/or large samples, which have been well documented in previous research [48,49]. Obviously, the large sample size of the present study can easily cause a significant χ2 value (as seen in the result section). Therefore, although reported, the χ2 statistics were not further discussed in considerable detail; instead greater emphasis was placed on the fit indices that supplement the χ2 statistic. Previous studies have shown that the CFI, and RMSEA statistics are less sensitive to sample size and could be recommended as alternative goodness-of-fit criteria that are superior to χ2 (or Δχ2) for testing invariance in large samples [44,48]; consequently these were emphasized in this study. Following recommendations by Chen (2007) for comparing two nested models, cut-off values of ΔCFI < 0.01 and ΔRMSEA < 0.015 were used for testing metric invariance, scalar invariance, as well as uniqueness invariance [48]. In the present study, models were considered acceptable on condition that both indices met the above criteria.

## Results

### Model fit for CES-D

Table 2 presents the goodness-of-fit indices for the four-factor, three-factor, single-factor, and second-order models of the CES-D in the Chinese and Dutch samples. The results indicated that the single-factor CFA models showed the worst fit to the data for both samples; they had the largest χ2 and RMSEA values, and lowest CFI, and NNFI values, although their RMSEA values were close to the cut-off value of 0.08. For both samples, the four-factor, second-order, and three-factor model adequately fit the data (i.e., CFI and GFI were larger than 0.90, RMSEA < 0.08, and SRMR < 0.06), and all item factor loadings were significant at the p < 0.05 level. Furthermore, the results indicated that the four-factor model fitted the data best in both samples, judging by all fit indices.

**Table 2:** Goodness-of-fit indices for models tested in the Chinese and Dutch sample

Model	χ^2^	df	AIC	CFI	NNFI	RMSEA
Chinese						
Four factor	2730.047	164	3009.371	0.979	0.976	0.058
Second order	5096.836	167	4753.926	0.959	0.954	0.074
Three factor	3129.081	167	3506.226	0.976	0.972	0.063
Single factor	7086.524	170	8032.134	0.943	0.936	0.096
Dutch						
Four factor	1209.586	164	1472.134	0.965	0.960	0.061
Second order	1983.275	167	2058.215	0.939	0.931	0.075
Three factor	1476.733	167	1774.343	0.956	0.950	0.068
Single factor	2381.659	170	2829.955	0.926	0.917	0.089
Invariance models						
Configural	3939.633	328	4481.504	0.976	0.972	0.059
Metric	4556.913	344	5092.937	0.061	0.969	0.062
Scalar	6775.092	360	7613.929	0.958	0.955	0.076
Partial Scalar	5351.410	358	5855.051	0.967	0.965	0.066
Uniqueness	7517.595	376	7295.681	0.953	0.952	0.073
Partial Uniqueness	6571.827	373	6682.538	0.959	0.958	0.070

Based on the above CFA results, reliability estimates for the 4 factors (subscales) were computed. Although internal consistency coefficient alpha is widely used as a reliability estimate, a number of problems arise from its use (e.g., alpha does not provide information about the internal structure of an instrument [50]). The omega coefficient is thought to be a better index for internal consistency [51]. Therefore, the omega coefficients of four factors were calculated for both samples. The results indicated that the omega coefficients of Somatic complaints, Depressive affect, Positive affect, and Interpersonal problems in the current Chinese sample were 0.811, 0.878, 0.725, and 0.722, respectively, and in the Dutch sample they were 0.746, 0.829, 0.755, and 0.570, respectively.

In subsequent analyses, the four-factor structure of the CES-D was used as a baseline model for testing measurement invariance across the Chinese and Dutch sample.

### Measurement Invariance

#### Configural invariance

The first test of configural invariance assessed whether the CES-D was best described by a four-factor structure for the two samples. The results showed that the configural invariance model fitted the data reasonably well, RMSEA = 0.059 (90% CI = 0.058, 0.061), CFI = 0.976 (other fit indices are reported in table 2). All factor loadings were significant (p < 0.05). These results indicate that the four-factor model fitted the data well in both samples.

#### Metric invariance

Following the configural invariant model, a metric invariance model was tested. To establish metric invariance, factor loadings were constrained to be equal across groups; intercepts and residual variances were freely estimated; and factor means were fixed to zero in both groups. The constrained model showed acceptable model fit, RMSEA = 0.062 (90% CI = 0.061, 0.064), CFI = 0.972 (other fit indices are reported in table 2). The changes in fit indices between the configural and metric invariant model were not significant, ΔCFI = 0.004 and ΔRMSEA = 0.003. Both ΔCFI and ΔRMSEA were smaller than the cut-off values. These results suggest that factor loadings were invariant across the Chinese and Dutch sample.

#### Scalar invariance

To establish scalar invariance, intercepts and factor loadings were constrained to be equal across the two groups; the residual variances were freely estimated; and factor means were set to zero in one group and free in the other. The results showed a deterioration of fit: RMSEA = 0.076 (90% CI = 0.074, 0.077), CFI = 0.958 (other fit indices are reported in table 2). The changes in fit indices between the metric and the scalar invariance model were significant, ΔCFI = 0.014, and ΔRMSEA = 0.0148, which suggests that scalar invariance cannot be established across the two groups.

To establish partial scalar invariance, we searched for items that were not invariant across groups. After repeating the procedure of searching for items that were not invariant several times, equality constraints were lifted for two items ("failure" and "good") on the Depressive Affect and Positive Affect factor. Results showed that the fit indices for the partial scalar invariance model were adequate: RMSEA = 0.066 (90% CI = 0.064, 0.07), CFI = 0.967 (see table 2 for the other fit indices). The changes in model fit indices between the metric invariance model and the partial scalar invariance model were no longer significant, ΔCFI = 0.007 (< 0.01), ΔRMSEA = 0.004 (< 0.01).

#### Uniqueness invariance

To establish uniqueness invariance, uniqueness, intercepts and factor loadings were constrained to be equal across two groups.

Because full scalar invariance was not supported, the uniqueness and intercepts of the items that were not invariant across two samples were not constrained to be equal across the two samples, whereas the uniqueness and intercepts of other items were held invariant [36]. The constrained model showed acceptable model fit: RMSEA = 0.073 (90% CI = 0.071, 0.074), CFI = 0.953 (see table 2 for the other fit indices). However, the change in CFI (ΔCFI = 0.014) between the partial scalar and the uniqueness invariance model was significant, suggesting that uniqueness invariance did not hold across the Chinese and Dutch sample.

To test whether partial uniqueness invariance could be obtained, the procedure for searching for items that were not invariant was repeated several times, and the equality constraint of item intercepts of three items (depressed, fearful, and dislike) were eventually lifted. The fit indices of the partial uniqueness invariance model showed better model fit: RMSEA = 0.070 (90% CI = 0.068, 0.071), CFI = 0.959 (see table 2 for the other fit indices). The changes in model fit indices between the partial uniqueness invariance model and the partial scalar invariance model were no longer significant, ΔCFI = 0.008 and ΔRMSEA = 0.004. See table 3 for factor loadings, intercepts and uniquenesses for each item.

**Table 3:** Factor loadings, uniquenesses and intercepts of the CES-D for both samples

	Items	Unstandardized loadings	Standardized loadings	Unstandardized intercepts	Standardized intercepts	Unstandardized uniquenesses	Standardized uniquenesses
		
		Chinese - Dutch	Chinese - Dutch	Chinese - Dutch	Chinese - Dutch	Chinese - Dutch	Chinese - Dutch
SOM	Bothered	(1.00 - 1.00)	(0.55 - 0.55)	(0.69 - 0.69)	(0.86 - 0.86)	(0.45 - 0.45)	(0.70 - 0.70)
	Appetite	(1.03 - 1.03)	(0.60 - 0.60)	(0.56 - 0.56)	(0.74 - 0.74)	(0.37 - 0.37)	(0.64 - 0.64)
	Mind	(1.18 - 1.18)	(0.64 - 0.64)	(0.78 - 0.78)	(0.95 - 0.95)	(0.39 - 0.39)	(0.59 - 0.59)
	Effort	(1.29 - 1.29)	(0.67 - 0.67)	(0.81 - 0.80)	(0.95 - 0.95)	(0.40 - 0.40)	(0.55 - 0.55)
	Sleep	(1.07 - 1.07)	(0.53 - 0.53)	(0.88 - 0.88)	(0.98 - 0.98)	(0.57 - 0.57)	(0.72 - 0.72)
	Talk	(1.02 - 1.02)	(0.54 - 0.54)	(0.74 - 0.74)	(0.87 - 0.87)	(0.51 - 0.51)	(0.71 - 0.71)
	Get Going	(1.26 - 1.26)	(0.68 - 0.68)	(0.70 - 0.70)	(0.85 - 0.85)	(0.37 - 0.37)	(0.54 - 0.54)

DEP	Blues	(1.00 - 1.00)	(0.69 - 0.69)	(0.52 - 0.52)	(0.70 - 0.70)	(0.29 - 0.29)	(0.53 - 0.53)
	Depressed	(1.13 - 1.13)	(0.75 - 0.75)	(0.55 - 0.55)	(0.72 - 0.72)	0.30 - 0.13	0.51 - 0.22
	Failure	(0.82 - 0.82)	(0.60 - 0.60)	0.57 - 0.30	0.80 - 0.43	0.41 - 0.09	0.83 - 0.17
	Fearful	(0.91 - 0.91)	(0.67 - 0.67)	(0.42 - 0.42)	(0.61 - 0.61)	0.31 - 0.14	0.64 - 0.29
	Lonely	(1.11 - 1.11)	(0.73 - 0.73)	(0.57 - 0.57)	(0.73 - 0.73)	(0.29 - 0.29)	(0.47 - 0.47)
	Crying	(0.92 - 0.92)	(0.68 - 0.68)	(0.50 - 0.50)	(0.70 - 0.70)	(0.27 - 0.27)	(0.54 - 0.54)
	Sad	(1.06 - 1.06)	(0.74 - 0.74)	(0.56 - 0.56)	(0.76 - 0.76)	(0.24 - 0.24)	(0.45 - 0.45)

POS	Good	(1.00 - 1.00)	(0.32 - 0.32)	1.54 - 0.68	1.41 - 0.62	1.20 - 0.72	1.01 - 0.60
	Hopeful	(1.66 - 1.66)	(0.54 - 0.54)	(1.36 - 1.36)	(1.28 - 1.28)	(0.81 - 0.81)	(0.71 - 0.71)
	Happy	(2.30 - 2.30)	(0.82 - 0.82)	(1.16 - 1.16)	(1.18 - 1.18)	(0.32 - 0.32)	(0.33 - 0.33)
	Enjoyed	(2.29 - 2.29)	(0.81 - 0.81)	(1.08 - 1.08)	(1.11 - 1.11)	(0.32 - 0.32)	(0.34 - 0.34)

INT	Unfriendly	(1.00 - 1.00)	(0.75 - 0.75)	(0.44 - 0.44)	(0.66 - 0.66)	(0.19 - 0.19)	(0.44 - 0.44)
	Dislike	(0.94 - 0.94)	(0.73 - 0.73)	(0.41 - 0.41)	(0.64 - 0.64)	0.23 - 0.08	0.57 - 0.19

Note. SOM = Somatic complaints, DEP = Depressive affect, POS = Positive affect, INT = Interpersonal problems. A bracketed value means that the equality restriction held for the Chinese and Dutch sample. Non-bracketed values indicate that the invariance constraint was lifted for that item.

### Latent Mean Difference

Based on the result of partial uniqueness invariance, comparison of latent factor mean differences across the Chinese and Dutch elderly groups was possible. Latent mean values were set to zero in the Chinese group and freely estimated for the Dutch group in the partial uniqueness invariance model, to assess latent mean differences. As expected, latent mean values in the Dutch group were significantly different from zero (all p's < 0.01). Results showed lower latent mean values for the Dutch group on all four dimensions of the CES-D. Means, standard deviations and effect sizes are presented in table 4. On average, Chinese elderly were more depressed than Dutch elderly, scoring about half a standard deviation higher on the latent traits. Standard deviations were larger in the Chinese sample as well, compared to the Dutch sample. The largest difference was found on the Interpersonal Problems factor (d=-0.650), and the smallest difference was found on the Positive Affect factor (d = -0.361).

**Table 4:** Latent mean differences

	Chinese		Dutch		d^c^
		
	M^a^	SD	M^b^	SD	
Somatic complaints	0.000	0.482	-0.261	0.324	-0.589

Depressive affect	0.000	0.570	-0.259	0.318	-0.506

Positive affect	0.000	0.354	-0.125	0.329	-0.361

Interpersonal problems	0.000	0.574	-0.323	0.184	-0.650

^a^. Latent means for the Chinese sample are fixed to zero.

^b^. Latent means for the Dutch group are freely estimated as deviations from the Chinese sample.

^c^. Effect size calculations are based on Hancock (2001); negative *d *values indicate higher scores for Chinese elderly adults.

## Discussion and Conclusions

### Factor Structure of the CES-D

The purpose of this study was to test the measurement invariance of the CES-D using confirmatory factor analysis in two large elderly populations from China and the Netherlands. The results reveal that in both samples, Radloff's four-factor model [4] resulted in a significantly better fit compared to a single-factor, three-factor, and second-order model. Hence, a model of four dimensions of the CES-D seems to be the most informative in assessing depressive symptoms in both the Chinese and Dutch elderly populations. This finding is consistent with a growing body of research comparing measurement models of the CES-D in various populations [9,10]. Our study extends the generalizability of this structure by replication in Chinese and Dutch elderly population-based samples. The twenty items of the CES-D can be interpreted in terms of four symptom dimensions including somatic complaints, depressed affect, positive affect, and interpersonal problems in both population groups. However, we could not replicate the factor structure suggested by earlier studies, in which the first-order factors are dependent on a single second-order factor [1,11,12].

### Measurement Invariance

Results obtained from the test of configural invariance confirmed the four factor structure across both samples. That is, both populations demonstrate equivalence in the pattern of factor loadings of the CES-D, suggesting that the CES-D measures the same concept across the Chinese and Dutch elderly. Our analysis also supported metric invariance across the two samples. This finding seems to imply that the twenty items of CES-D measure depressive symptoms (or depressed affects) in the same way across the two national samples. According to the interpretation of factor loadings suggested by Oort (2005) [52], reported feelings of the twenty items (e.g., bothered, depressed, and sadness) seem to be equally indicative of the four factors of the CES-D among the Chinese and Dutch elderly.

At the intercept level, full invariance was not supported. Two intercepts in the Depressed Affect factor (failure) and Positive Affect factor (good) differed across the Chinese and Dutch elderly. Specifically, the intercepts for failure and good were larger in the Chinese sample, which indicates a difference in internal standards across the Chinese and Dutch elderly [52]. Chinese elderly seem more inclined to endorse failure (Depressed Affect) and good (Positive Affect), compared to Dutch elderly with the same latent trait score.

Our analysis also did not support full uniqueness invariance across the two samples. The partial invariance analysis revealed that the Depressed Affect and Interpersonal Problems domain of the CES-D is less invariant than the other two domains. Specifically, the invariance of *depressed *and *fearful *on the Depressed Affect factor and *dislike *in the Interpersonal Problems factor did not hold across the two samples. Uniquenesses of *depressed, fearful *and *dislike *were larger in the Chinese sample, suggesting that the items' measurement errors were larger for Chinese elderly adults than for Dutch elderly adults.

The differences in intercepts and uniquenesses of these items may result from the cultural differences and differing social norms, which could influence the way one experiences and expresses feelings of depression. For example, Nikelly (1998) suggested that the expression of affective distress causes the individual to appear self-centered, which may be threatening to close relationships and therefore discouraged in collective cultures [25]. In addition, the stigma surrounding mental illness in Chinese culture could also preclude the expression of depressed affects [26]. As a result, depressed affect is more likely to be devalued in Chinese culture, so somatic symptoms may constitute a more expedient means to express depressive symptoms than depressed affect for the Chinese population [30,53]. Such differences between Eastern and Western cultures could explain why the invariance restriction did not hold for some items. However, we should be careful in using cultural differences to interpret each loading or intercept difference of items which are not invariant, as it is hard to disentangle the contents of cultures and the specific psychological process that differ across countries and that could explain the supposed cultural differences [54].

Although only partial metric and scalar invariance were supported, the meaningful comparison of factor means of the CES-D across the Chinese and Dutch elderly seems possible. Cheung and Rensvold (1998, 1999) suggested that if the proportion of non-invariant items of a scale is small, the comparison of factor means can still be meaningful even if full measurement invariance does not hold, as the non-invariant items will not heavily affect the comparison [55,56]. Therefore, the cross-country comparison of the four-factor means of the CES-D could be meaningful. However, the estimated factor mean difference may be different depending on the anchor items selected for the factor [57]. When comparing mean values of some dimensions (or some items) of the CES-D between the Chinese and Dutch elderly, the differences between intercepts for the two items of the Depressive Affect- and Positive affect- factor, and uniquenesses for the three items of the Depressive Affect- and Interpersonal Problems- factor, should be taken into account through latent variable methodologies.

### Latent mean differences

Latent mean differences between the Chinese and Dutch sample were found on all four CES-D factors, with the Dutch scoring about half a standard deviation lower than the Chinese elderly. This indicates that, on average, the Chinese elderly reported more feelings of depression than the Dutch elderly.

### Implications and Future Directions

The current study has two implications. First, based on the number of previous studies on the psychometric properties of the CES-D [9,12], the present study takes a further step in understanding the internal validity of the CES-D, confirming its four-factor structure and demonstrating its generalization to a typically Western and a typically non-Western country. Second, results obtained from this study have significant implications for studies comparing the depressive symptoms between Chinese and Dutch elderly using the CES-D. We have established configural invariance and metric invariance for the CES-D across the two national groups. This implies that the CES-D measures the same concept across the Chinese and Dutch elderly. Partial scalar invariance and partial uniqueness invariance were also established, indicating that comparisons of the factor means of CES-D may be meaningful between Chinese elderly and Dutch elderly groups to some extent, although there were some differences in item intercepts and uniquenesses.

There are several limitations to the current study. First, only the equivalence of factor validity was studied. This is insufficient to demonstrate that it is an effective measurement both for populations from two countries. A goal for future research is to examine whether the other types of validity, such as predictive concurrent and content validity of the CES-D are also equivalent across the two population groups. Second, although China and the Netherlands serve as examples of countries with different social and cultural backgrounds in the current study, future studies should be conducted using samples from other typically Western and Eastern countries to see whether the results can be replicated, in order to demonstrate the generalization of the CES-D across different cultural backgrounds. Third, when interpreting the loading or intercept differences of non-invariant items, caution should be applied because of chance capitalization. Releasing parameter restrictions based on modification indices and expected change is a data driven procedure, and susceptible to capitalization on chance characteristics of the data [58]. The model modifications we applied to obtain partial measurement invariance should be replicated, to ascertain the generalizability of our results as well.

### Appendix. English, Chinese, and Dutch versions of CES-D

#### English version of the CES-D

01. I was bothered by things that usually don't bother me.

02. I did not feel like eating; my appetite was poor.

03. I felt that I could not shake off the blues even with the help of my family or friends.

04. I felt I was just as good as other people.

05. I had trouble keeping my mind on what I was doing.

06. I felt depressed.

07. I felt that everything I did was an effort.

08. I felt hopeful about the future.

09. I thought my life had been a failure.

10. I felt fearful.

11. My sleep was restless.

12. I was happy.

13. I talked less than usual.

14. I felt lonely.

15. People were unfriendly.

16. I enjoyed life.

17. I had crying spells.

18. I felt sad.

19. I felt that people disliked me.

20. I could not get "going."

#### Chinese version of the CES-D

01. 我最近烦一些原来不烦心的事

02. 我不想吃东西，胃口不好

03. 我觉得沮丧，就算有家人和朋友帮助也不管用

04. 我觉得自己和别人一样好

05. 我不能集中精力做事

06. 我感到消沉

07. 我觉得做每件事都费力

08. 我感到未来有希望

09. 我觉得一直以来都很失败

10. 我感到害怕

11. 我睡不安稳

12. 我感到快乐

13. 我讲话比平时少

14. 我觉得孤独

15. 我觉得人们对我不友好

16. 我生活愉快

17. 我哭过或想哭

18. 我感到悲伤难

19. 我觉得别人不喜欢我

20. 我提不起劲儿来做事

#### Dutch version of the CES-D

01. De afgelopen week maakte ik me zorgen om dingen waar ik me anders geen zorgen over maak.

02. De afgelopen week had ik geen zin in eten, was mijn eetlust slecht.

03. De afgelopen week kon ik een neerslachtige stemming niet van me afschudden, zelfs niet met behulp van mijn familie en vrienden.

04. De afgelopen week voelde ik me evenveel waard als andere mensen.

05. De afgelopen week had ik moeite mijn gedachten te houden bij wat ik aan het doen was.

06. De afgelopen week voelde ik me depressief.

07. De afgelopen week had ik het gevoel dat alles wat ik deed me moeite kostte.

08. De afgelopen week was ik hoopvol gestemd over de toekomst.

09. De afgelopen week vond ik mijn leven een mislukking.

10. De afgelopen week voelde ik me angstig.

11. De afgelopen week had ik een onrustige slaap.

12. De afgelopen week was ik gelukkig.

13. De afgelopen week praatte ik minder dan gewoonlijk.

14. De afgelopen week voelde ik me eenzaam.

15. De afgelopen week waren de mensen onvriendelijk.

16. De afgelopen week had ik plezier in het leven.

17. De afgelopen week moest ik soms huilen.

18. De afgelopen week voelde ik me bedroefd.

19. De afgelopen week had ik het gevoel dat de mensen me niet aardig vonden.

20. De afgelopen week kon ik maar niet goed op gang komen.

## Competing interests

The authors declare that they have no competing interests.

## Authors' contributions

BZ designed the protocol, analyzed the data and prepared the manuscript. MF conceived the study, participated in the study design and gave significant comments on the manuscript. PC helped to conceive the study and gave his significant comments for improving the manuscript. JL had full access to the Chinese data in the study, helped to design the protocol and draft the manuscript, and took responsibility for the integrity of the data. NS helped to conceive the study and gave significant comments for improving the manuscript. AB had full access to the LASA data and gave significant comments on the manuscript.

All authors have read and approved the final version of the manuscript.

## Pre-publication history

The pre-publication history for this paper can be accessed here:

http://www.biomedcentral.com/1471-2288/11/74/prepub
